# Unveiling 2,000 years of differentiation among Tungusic-speaking populations: a revised phylogeny of the paternal founder lineage C2a-M48-SK1061

**DOI:** 10.3389/fgene.2023.1243730

**Published:** 2023-07-24

**Authors:** Hui-Xin Yu, Cheligeer Ao, Xian-Peng Zhang, Kai-Jun Liu, Yi-Bing Wang, Song-Lin Meng, Hui Li, Lan-Hai Wei, Da Man

**Affiliations:** ^1^ Institute of Anthropology and Human Sciences, School of Ethnology and Anthropology, Inner Mongolia Normal University, Hohhot, China; ^2^ Chengdu 23Mofang Biotechnology Co., Ltd., Chengdu, China; ^3^ School of History and Ethnic Culture, Hulunbuir University, Hulunbuir, China; ^4^ MOE Key Laboratory of Contemporary Anthropology, School of Life Sciences, Fudan University, Shanghai, China; ^5^ B&R International Joint Laboratory for Eurasian Anthropology, Fudan University, Shanghai, China; ^6^ College of Life Science and Technology, Inner Mongolia Normal University, Hohhot, China

**Keywords:** Tungusic-speaking populations, haplogroup C2a-M48-SK1061, founder paternal lineage, phylogeny, human evolution

## Abstract

Previous studies demonstrated Y chromosome haplogroup C2a-M48-SK1061 is the only founding paternal lineage of all Tungusic-speaking populations. To infer the differentiation history of these populations, we studied more sequences and constructed downstream structure of haplogroup C2a-M48-SK1061 with better resolution. In this study, we generated 100 new sequences and co-analyzed 140 sequences of C2a-M48-SK1061 to reconstruct a highly revised phylogenetic tree with age estimates. We also performed the analysis of the geographical distribution and spatial autocorrelation of sub-branches. Dozens of new sub-branches were discovered, many sub-branches were nearly unique for Ewenki, Evens, Oroqen, Xibe, Manchu, Daur, and Mongolian. The topology of these unique sub-branches is the key evidence for understanding the complex evolutionary relationship between different Tungusic-speaking populations. The revised phylogeny provided a clear pattern for the differentiation history of haplogroup C2a-M48-SK1061 in the past 2,000 years. This study showed that the divergence pattern of founder lineage is essential to understanding the differentiation history of populations.

## 1 Introduction

The Tungusic-speaking population mainly distributed in north Asia, their evolutionary history, unique subsistence patterns, and cultural traditions has long fascinated linguists, archaeologists, and geneticists, and anthropologists. Previous archaeological studies indicated that the rise and prosperity of the Uril, Poltse and Mohe cultures were directly related to the development of Tungusic-speaking populations (Twitchett and Franke, 1994; Elliott, 2001; Feng, 2002; Gorelova, 2002; Derevianko, 2015; Diakova, 2016). Most genetic studies of Asians involve Tungusic speakers. As a result, previous studies indicated that haplogroup C2a-M86 was the only founding paternal lineage because it occurred so frequently in paternal makeup of across different Tungusic speakers (Karafet et al., 2001; Lell et al., 2002; Zerjal et al., 2002; Puzyrev et al., 2003; Derenko et al., 2006; Hammer et al., 2006; Pakendorf et al., 2006; Xue et al., 2006; Khar’kov et al., 2008; Malyarchuk et al., 2010; Zhong et al., 2010; Duggan et al., 2013; Malyarchuk et al., 2013; Karmin et al., 2015; Pugach et al., 2016; Karafet et al., 2018; Balinova et al., 2019). Moreover, there is higher diversity of paternal lineage; haplogroup N, Q, R, and O also can be found in Tungusic-speaking populations, which may be attributed to the population admixture in history. In our previous preliminary study of downstream lineages of M86, we found SK1061 is the specific paternal lineage of Tungusic speakers, and revealed multiple important sub-clades of SK1061 (Liu et al., 2020).

It is generally accepted that the diversification of language is closely related to the differentiation of populations (Bellwood and Renfrew, 2002). While previous studies indicated multiple specific paternal lineages could be found in populations from different linguistic groups (Karafet et al., 2001), Tungusic-speaking populations all share only one founding paternal lineage (SK1061). Therefore, we speculated that the differentiation of founding paternal lineage would be directly associated with diversity of Tungusic speakers, and the topology structure of founding paternal lineage is consistent with the diversified structure of the Tungusic language (Wei and Li, 2017). However, no existing studies confirm our hypothesis.

In this study, we analyzed 140 samples belonging to haplogroup SK1061, and we had three goals: 1) to build a higher-resolution revised phylogenetic tree, 2) to investigate whether there are specific paternal haplogroups in different Tungusic speakers, and if so, to chart the topological structure of these sub-branches, and 3) to explore the relationship between the phylogenetic structure of the founding paternal lineage and patterns of population diversification, shifts of archaeological culture, and development of language genealogy.

## 2 Materials and methods

**Samples and Sequencing** Saliva samples were collected from unrelated healthy males in East Asian populations over the last few decades. All the participants provided written informed consent prior to participating. The study and sample collection process were reviewed and approved by the Medical Ethics Committee of Fudan University and Inner Mongolian Normal University, and complied with the ethical principles of the 2013 Helsinki Declaration of the World Medical Association. Genomic DNA was extracted using the DP-318 Kit (Tiangen Biotechnology, Beijing) according to the manufacturer’s protocol. Firstly, Routine Y-SNP tests were done to determine the general haplogroup of all male samples. And then DNA specimens extracted from C2a-M48-SK1061 samples were sent for next-generation sequencing on the Illumina HiSeq2000 platform (Illumina, San Diego, CA, United States).

**Data Analysis** In total, 140 sequences of SK1061 individuals were analyzed (Supplementary Table S1). We used the procedure that we described previously for the other steps prior to next-generation sequencing, i.e., for DNA shearing, adding an adaptor, and gel electrophoresis (Sun et al., 2021). Read mapping and SNP calling from next-generation sequencing data were conducted using standard procedures (BWA and Samtools) and the human reference genome sequence, hg38 (Li and Durbin, 2009; Li et al., 2009; Li and Durbin, 2010). To obtain a confident Y-SNP dataset for reconstruction of phylogenetic tree and age estimation, we applied a series of strict filters on the original variants file, including: 1, restriction to variants that are single nucleotide polymorphisms (Y-SNP); 2, removal of all positions with call rate <80% on all samples with sequences; 3, removal of position with heterozygosis call rate >5% on all samples; 4, base coverage ≥3, base quality >20, and distance between SNPs >10 bp; 5, removal of recurrent or triadic mutations. The regulations proposed by the YCC were followed to revise the phylogenetic tree with respect to new variants in the non-recombining region of the Y chromosome (Y Chromosome Consortium, 2002). Since there is the phylogenetic tree of C2a-M48-SK1061 in previous studies, the revised phylogenetic tree was constructed based on previous studies, public resources, and the genetic genealogy community. Bayesian evolutionary analyses were conducted using BEAST (v.2.0.0) (Bouckaert and Drummond, 2017). To calculate divergence times in the phylogenetic tree, a point mutation rate of 0.74 × 10–9 per site per year (Karmin et al., 2015), inferred from the ∼12,000-year-old Anzick-1 male infant genome (Rasmussen et al., 2014), was applied. New haplogroup names for sub-branches of C2a-M48-SK1061 are listed in Supplementary Table S1.

## 3 Results

**FIGURE 1:**
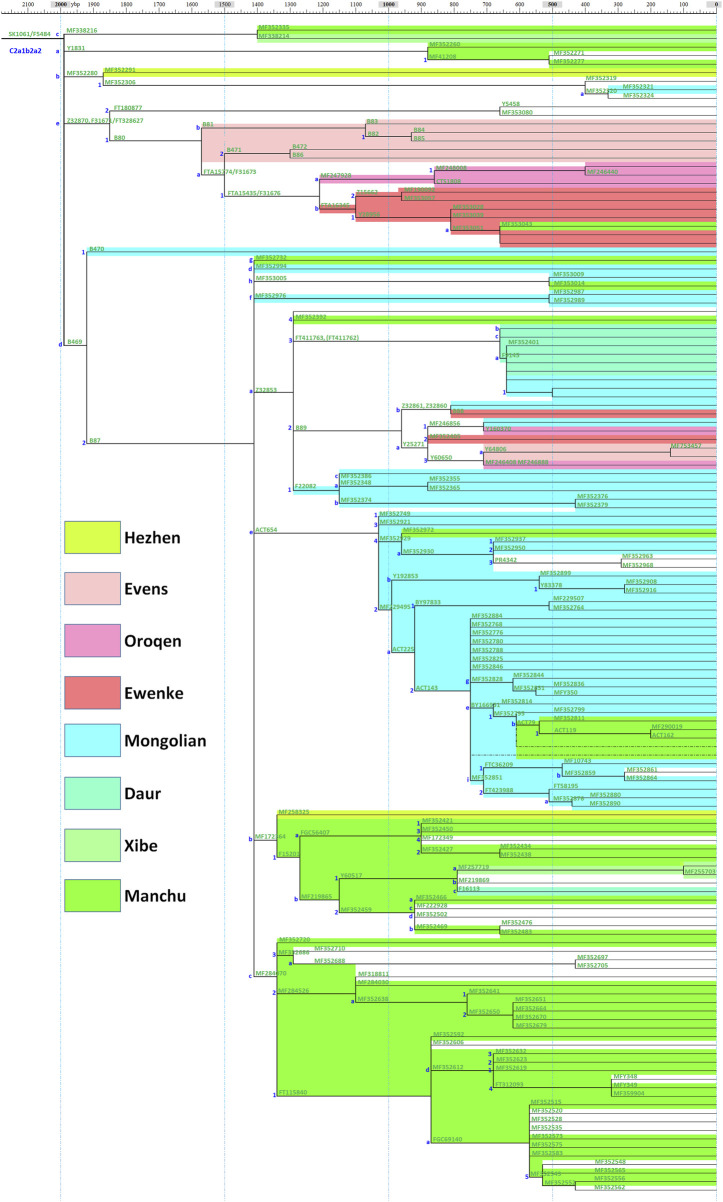
Schematic diagram of the revised phylogeny of haplogroup C2a-M48-SK1061.

**FIGURE 2:**
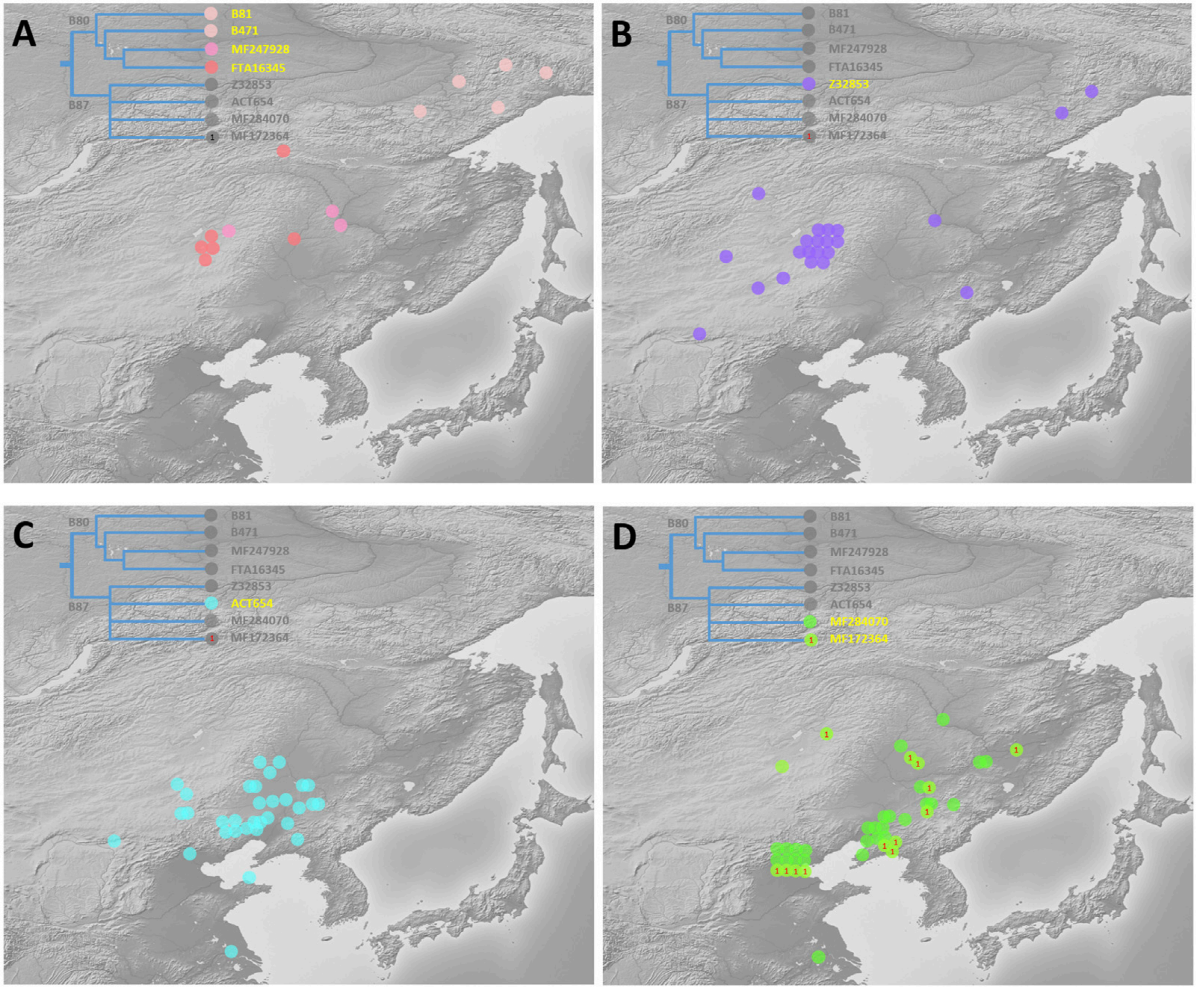
Geographic distribution of eight main sub-branches of C2a-M48-SK1061 **(A)**: paternal lineage B80, which is unique for Even, Ewenki, and Oroqen, mainly distributed in high altitude in northern Asia. **(B)** Z232853 distributed mainly in the border zone between Northeast China and Russia. **(C)** ACT654 occurred mainly in Mongolic speakers in southwestern region of Northeast China. **(D)** MF284070 and MF172346 mainly distributed in Northeast China and Beijing).

A schematic representation of the revised phylogeny of haplogroup C2a-M48-SK1061 with age estimation is shown in Figure 1. The detailed tree can be found in Supplementary Table S1. We detected a complex downstream structure and identified numerous new SNPs and sub-branches. The geographic distribution of eight main clades of SK1061 is shown in Figure 2. Additionally, we found dozens of new downstream clades and provided a chronology for each of these clades (Figure 1; Supplementary Table S1). Overall, the differentiation time of SK1061 in Tungusic-speaking populations is about 2,000 years ago (kya). In the past 2,000 years (ky), haplogroup SK1061 expanded continually and generated many sub-branches, including several that developed independently and formed specific paternal lineage in different populations.

In this study, we found a number of specific paternal lineages and haplogroup classifications (Figure 1; Supplementary Table S1). Haplogroup B81 and B471 are the specific paternal lineages of Evens, haplogroup F31676 is the specific paternal lineage of Ewenki and Oroqen, MF247928 is the specific paternal lineage of Oroqen, and FTA16345 is the specific paternal lineage of Ewenki. The divergence time of Ewenki and Oroqen is about 1.2 kya, and the divergence time between Evens and Ewenki and Oroqen is around 1.5 kya which we determined by observing the revised phylogenetic tree with an age estimate. We found the paternal lineage of a common ancestor group of Evens, Ewenki and Oroqen is Z32870, which can be considered the northern branch of SK1061, and the differentiation time of Z32870 is about 1.6 kya.

Based on the phylogenetic structure, we found a dramatic expansion in paternal lineage B87 about 1.4 kya and generated eight main sub-branches (Figure 1; Supplementary Table S1). Evens, Ewenki and Oroqen share the paternal lineage B89, Mongolian and Daur share FT411763 and F20822, and B89, FT411763 and F20822 are the sub-clades of Z32853. Interestingly, we found a more prosperous paternal lineage in Mongolic speakers from eastern China: ACT654. The differentiation time of this branch is about 1.0 kya including a large-scale expansion about 0.8 kya. However, ACT654 is unique for Mongolic speakers in modern populations.

As shown on Figure, MF172364 and MF284070 are two large clades that can be found in Manchu, Hezhen and Xibe, and the differentiation time of these branches is 1.4 kya. Based on the phylogenetic tree, we found some Han people appeared on the sub-clades that are nearly specific to Manchu, Hezhen and Xibe. In general, we proposed that these two clades may be key paternal lineages of Wuji, Mohe, Jurchen, and Manchu populations.

## 4 Discussion

### 4.1 Relationship between topological structure of SK1061 and population differentiation

**FIGURE 3:**
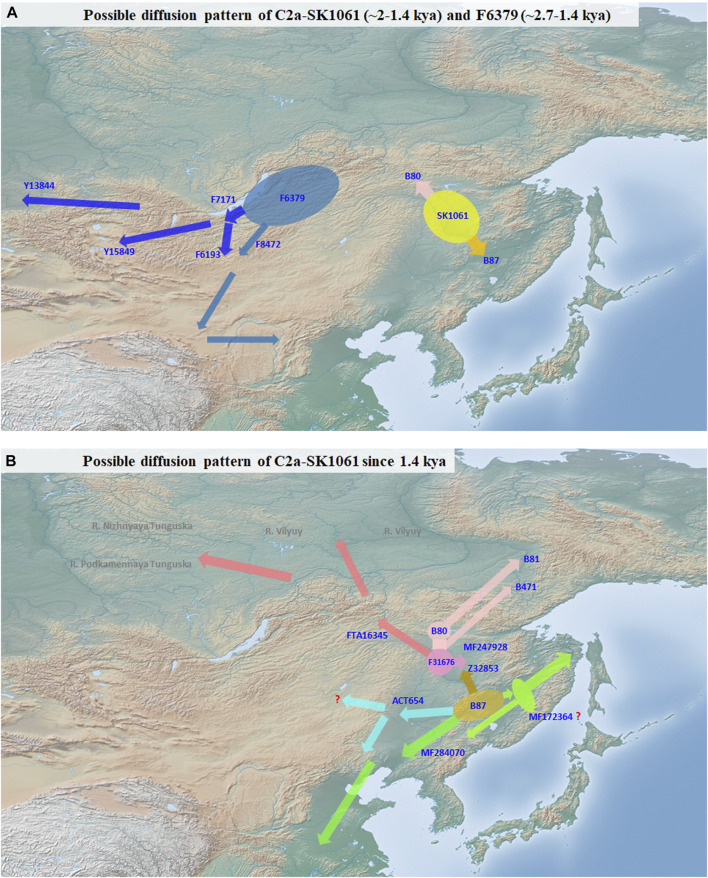
Possible diffusion pattern of C2a-M48-SK1061. **(A)**: possible diffusion pattern of C2a-M48-SK1061 (∼2-1.4 kya) and F6379 (∼2.7-1.4 kya). **(B)**: possible diffusion pattern of C2a-M48-SK1061 since 1.4 kya.

Our study exhibits the divergence and expansion pattern of SK1061 in the past 2 ky (Figure 3). We found that the topological structure of SK1061 is related to the differentiation pattern of Tungusic speakers and proposed that differentiation pattern of SK1061 is consistent with the diversification history of Tungusic language. We also identified the specific paternal lineage of different Tungusic speakers. For example, B80, which is unique to the Evens, Ewenki and Oroqen, diverged from SK1061 about 2 kya. By contrast, B87, which is the main paternal lineage of southern Tungusic speakers such as the Manchu, Hezhen and Xibe, is generated concurrently. In general, the phylogenetic structure supported the north-south structure of Tungusic-speaking populations.

According to history records and investigation of anthropology, the differentiation between Evens and Ewenki is about 1.5 kya (Shirokogoroff, 1924, 1966). In this study, we found the differentiation time of B80 is 1.5 kya, and there is a consistency between diverged time in memory of Evens and Ewenki and differentiation time of paternal lineage (Figures 1, 3). In addition, the Oroqen, who inhabited the eastern region of the Zeya River Basin since at least the 15th century, be considered as an eastern group of Ewenki in the historical record (Shirokogoroff, 1966). We found MF247928 is not only unique for the Oroqen, but also that it is close with FTA16345 which can be found in the Ewenki, and further confirmed historical record.

Lineage Z32853 is a distinctive sub-branch of B87. Based on sample information of other sub-clades of B87, we believed B87 may be a key paternal lineage of the Mohe people distributed across the middle and lower parts of the Amur River Basin (Figure 3). However, Z32853 mainly appeared in the Mongolian and Daur people in northeast China, and the expansion time of this lineage is less 1.3 kya (Figures 1, 3). Therefore, we propose the formation of Z32853 to be related to the historical northwestern movement of Tungusic speakers around that time. After that point, the Z32853 became continually differentiated in the upper and middle Amur River Basin. Serval lineages flowed into Evens, Ewenki, and Oroqen, but FT411763 integrated into the Daur and other populations in the Hulunbuir region while F22082 appeared only in Mongolians. In general, the differentiation and distribution patterns of the Z32853 revealed the admixture history between Mongolic speakers and the Tungusic-speaking populations who migrated into the upper Amur River Basin.

We also found that ACT654 mainly occurred in Mongolian people in northeast China as well as Manchu people who may have mixed with Mongolians (Figure 3). Based on our findings, we also believe that lineage ACT654 may be the major paternal lineage of western part of the ancient Wuji and Mohe populations. These peoples had previously settled in the middle and lower parts of the Amur River Basin before migrating to western regions of northeastern China such as Daxing’anling and Nenjiang River during the Song and Jin dynasties. The large-scale and continuous expansion of ACT654 occurred around the same time 0.8 kya as the rise of the Mongolian empire and the establishment of the Yuan dynasty. During this history period, ancient people with ACT654 may had become parts of Mongolian. Overall, the appearance of ACT654 in eastern parts of Mongolian may be results of population admixture in the past one thousand years.

Furthermore, we propose that MF172364 and MF284070 are the key paternal lineages of the Wuji, Mohe, Jurchen, and Manchu people (Figure 3). These branches maybe generated from B87 after people whose main paternal lineage is B87 migrated southward from the middle Amur River Basin. Moreover, several small sub-lineages were formed simultaneously including MF338216, Y1831, and MF352392. MF172364 and MF284070 expanded continually from 1.4 ky, which is consistent with the demographic history of the Mohe people, and these lineages also appeared in modern Manchu peoples, thus supporting the historical development from Wuji to Mohe to Jurchen to Manchu. In addition, we found that there are several specific surnames of Manchu people who belonged to either MF172364 or MF284070 (Figure 3). According to the historical record, after Mohe empire was established, they accepted the rule of the Tang dynasty (Liu, 2009; Derevianko, 2015). This historical account coincides with the differentiation time and geographic distribution of B87, MF172364, and MF284070. A previous study has indicated Mohe people contributed up to 75%–81.2% genetic ancestry to modern Manchu people inferred from genome-wide data (Zhang et al., 2021), and ancient Mohe individual had an estimated 43% ± 15% Amur River Basin Neolithic ancestry (Wang et al., 2021). In general, we believe that B87 may be the paternal lineage of a leader of the Heishui Mohe. Finally, we explored the diffusion process of the mostly close clade of SK1061, F6379. Based on topological structure and geographic distribution, we believe the origin of F6379 is in the Transbaikal region, and that it expanded toward the central region of the Mongolian plateau in later period.

### 4.2 Correlation between patrilineal differentiation and archaeological culture

The differentiation time of SK1061 is about 2.0 kya, which is after the period when the Uril culture (∼3,300–2,400 BP) existed (Feng, 2002). We speculated that the Uril culture might have been established by the ancestors of Tungusic speakers. The Poltse culture distributed in the middle and lower parts of the Amur River Basin, and the Talakan culture distributed in the middle and upper parts of the Amur River Basin (Feng, 2002). These two ancient cultures showed an east-west dichotomy pattern. Therefore, we believe that the distribution of the two cultures corresponded to the north-south structure of two sub-branches of SK1061 (Z32870 vs B87). As a result, we propose B87 as the dominant paternal lineage of the Wuji and Mohe peoples, though further evidence of ancient DNA is needed to confirm our hypothesis.

### 4.3 Correlation between patrilineal differentiation and language genealogy

In this study, we claim that the phylogenetic tree of SK1061, which is the founding paternal lineage of Tungusic speakers, is helpful for exploring differentiation, kinship, and genealogical relationship of sub-groups of the Tungusic language group. Our findings in this study confirm the consensus opinion among linguists that the Tungusic language can be divided into northern and southern branches (Doerfer, 1978; Vovin, 2009). In addition, the Manchu language has been considered distinct from other Tungusic languages and thus that Manchu speaker divided separately into the southern branch of the Tungusic language (Doerfer, 1978). However, this view was incorrect and has been corrected by linguists (Vovin, 2009). In this study, we found the main paternal lineage of Manchu people belonged to B87, the manjor paternal lineage of all southern Tungusic speakers. Overall, our results support the conclusion of linguists: Manchu and Jurchen speakers cannot be grouped separately into the southern branch of the Tungusic language. Instead, they form a southern branch with Ulchi and Udihe people. Interestingly, we found that a sample from the Xibe people showed a close relationship with Manchu. Furthermore, the phylogenetic structure of a Hezhen sample supported divergent structure from the southern branch of the Tungusic language. Nevertheless, we cannot make a conclusion due to the limited number of samples. In addition, in this work, the people who distributed in the lower Amur River Basin, such as the Negidal, Oroch and Orok peoples, are not included. Therefore, further study is needed.

In this study, we constructed a higher-resolution phylogenetic tree of C2a-M48-SK1061 by using more DNA sequences. We identified many specific paternal lineages of different Tungusic-speaking peoples and demonstrated the topological structure in the revised phylogenetic tree. In addition, we explored the 2,000-year differentiated history of Tungusic-speaking populations and established a relationship between different paternal lineages and archaeological cultures. Finally, we discussed the relationship between paternal topological structure and linguistic structure within Tungusic speakers. In conclusion, we propose differentiation, topological structure, and divergent patterns of founding paternal lineage to be related with population evolution and developmental history. However, further study is needed to show a more detailed relationship between the peoples.

## Data Availability

The original contributions presented in the study are included in the article/Supplementary Material, further inquiries can be directed to the corresponding authors.
